# The association between oxidative balance score and lung function: A nationwide cross-sectional survey

**DOI:** 10.1016/j.heliyon.2023.e14650

**Published:** 2023-03-18

**Authors:** Yoo Jeong Lee, In Cheol Hwang, Hong Yup Ahn

**Affiliations:** a Department of Family Medicine, Korea University Guro Hospital, Seoul, Republic of Korea; b Department of Family Medicine, Gil Medical Center, Gachon University College of Medicine, Incheon, Republic of Korea; c Department of Statistics, Dongguk University, Seoul, Republic of Korea

**Keywords:** Cross-sectional study, Oxidative stress, Population surveillance, Respiratory function tests

## Abstract

**Purpose:**

Given the impact of oxidative imbalance on the development of airway pathologies, this study was undertaken to investigate the association between oxidative balance (OB) scores and lung function in the adult Korean population.

**Methods:**

Data of 17,368 adults with available OB scores and pulmonary function test results were extracted from the 2013–2019 Korean National Health and Nutrition Examination Surveys. Multivariable logistic regression models were used to calculate the odds ratios (ORs) and 95% confidence intervals (CIs) for reduced forced expiratory volume in 1 s (FEV_1_) and forced vital capacity (FVC) per 1-point decrease in OB score. Dose dependent association of reduced lung function with OB scores was also investigated.

**Results:**

Males, low-income subjects, individuals with comorbidities, and those with reduced pulmonary function had lower OB scores (representing oxidative balance). Overall, the association between oxidative imbalance and reduced lung function was remarkable in FVC than FEV_1_ (OR [95% CI], 1.06 [1.04–1.07] vs. 1.03 [1.02–1.04]; both p < 0.001). Linear relationships between the level of reduced lung function and OB scores were significantly noted (p for trend<0.001 in both FEV_1_ and FVC).

**Conclusion:**

Our findings suggest that oxidative imbalance is associated with reduced pulmonary function.

## 1 Introduction

Oxidative stress refers to a state in which pro-oxidants overcome the scavenging capacity of antioxidants. Such excessive and/or uncontrolled oxidative stress can damage human cells and organ systems through the influence on biomacromolecules, such as lipids, proteins, carbohydrates, and DNA. To prevent or stop those pathological process, a complex antioxidant system works: dietary and endogenous enzymatic and nonenzymatic antioxidants counteract the detrimental effect of oxidative stress by various mechanisms [1]. There have been an enormous variety and range of pro-oxidant and antioxidant enzymes and compounds [2], among them, the oxidative balance (OB) scoring system [3] was devised to semi-quantitatively measure oxidative stress based on nutritional and lifestyle assessments. To date, several studies have evaluated the association between OB scores and various health outcomes [4].

As the lungs are constantly exposed to atmospheric oxygen, they are susceptible to oxidative injury from myriad forms of reactive oxygen species and free radicals. Indeed, oxidative imbalance is thought to be involved in the development of many airway pathologies [5, 6]. Patients with idiopathic pulmonary fibrosis are in a state of profound oxidative stress and have a lower level of antioxidative potency compared to healthy individuals [7], which is equally true to those with chronic obstructive pulmonary disease [8].

However, the association between oxidative imbalance and lung function in the general population has received little research attention; the generalizability of a prior Italian study was limited by small sample size [9]. If a certain relation is present even before the overt lung disease, interventions would be warranted and justified given the cost-effectiveness of primary prevention for lung diseases [10]. In this study, we investigated the relationship between OB scores and spirometry results, using data representative of the Korean adult population.

## 2 Methods

### 2.1 Design and participants

The data used in this study was extracted from the Korean National Health and Nutrition Examination Survey (KNHANES 2013–2019) results. KNHANES is a nationwide population-based survey conducted annually by the Korean Ministry of Health and Welfare to assess the health status of the Korean population and to monitor trends in health risk factors. Subjects are randomly selected using a stratified, multistage, probability-based sampling design. KNHANES collects many variables including participants' demographic, social, health and nutritional status from three component surveys: the health interview, health examinations and nutritional survey. The health interview and health examination surveys are conducted over 3 days at mobile examination centers, which travel to locations across the country. The nutrition survey is conducted at participants’ homes a week after the health interview. Interviews and health examinations were performed using calibrated equipment and a standardized protocol. Detailed information on KNHANES is provided in a previous publication [11].

Initially, we identified 20,909 subjects aged 40–79 years old with available pulmonary function test data. Pulmonary function test was conducted in subjects with 40 years or older, and the age was not differentiated for subjects more than 80 years old (all were coded as “80”). After excluding subjects without available OB score, data on 17,368 individuals were included in the final analysis.

The database used in this study is publicly available, and microdata and analytical guidelines can be downloaded from the KNHANES website (https://knhanes.kdca.go.kr/knhanes/eng/index.do). Selected candidates were informed that they could refuse to participate in KNHANES without prejudice in accordance with the requirements of the National Health Enhancement Act. All participants provided written informed consent, and researchers followed the guidelines set forth in the Declaration of Helsinki. The Institutional Review Board (IRB) of Gachon University Gil Medical Center (IRB no. GFIRB2022-099) approved the study protocol.

### 2.2 Measures

Pre-bronchodilator spirometry was performed by trained technicians using a dry rolling seal spirometer (Model 2130, Sensor Medics Corporation, Yorba Linda, CA, USA), as per the criteria established by the American Thoracic Society/European Respiratory Society for standardization [12]. For each subject, we recorded the predicted value (%) for forced expiratory volume in 1 s (FEV_1_) and forced vital capacity (FVC). Data were carefully reviewed for acceptability, repeatability, and quality.

OB scores were determined for 10 *a priori* defined components related to oxidative stress, that is, 5 pro-oxidant factors (polyunsaturated fatty acids, n-6 fatty acids, smoking, alcohol, and obesity) and 5 antioxidant factors (carotene, retinol, vitamin C, n-3 fatty acids, and physical activity), which is similar to previous research [13]. Dietary intake was assessed by the 24 h recall method, which has been shown to reflect recent dietary intake. During the nutritional survey, trained interviewers investigated all food products ingested during the previous 24 h and recorded dietary information on timings, types of food, amounts, and cooking methods. The self-administered questionnaire completed by participants included smoking status, alcohol use, and physical activity. Dietary factors and physical activity (assessed using metabolic equivalents of tasks) were classified into quartiles of study cohort distributions, and the categorizations for lifestyles were as Table 1. Overall OB scores were calculated by summing individual factor scores; the maximum possible score was 30 points, and higher scores indicated antioxidant predominance.

**Table 1:** Oxidative balance score calculation.

	Assigned score			
Factors	0	1	2	3
*Dietary*				
PUFA (g)	Quartile 4 (≥13.4)	Quartile 3 (8.3–13.3)	Quartile 2 (4.8–8.2)	Quartile 1 (<4.8)
n-6 fatty acid (g)	Quartile 4 (≥11.2)	Quartile 3 (6.8–11.1)	Quartile 2 (3.9–6.7)	Quartile 1 (<3.9)
ß-carotene (μg)	Quartile 1 (<1,263)	Quartile 2 (1,263–2,353)	Quartile 3 (2,354–4,088)	Quartile 4 (≥4,089)
Retinol (μg)	Quartile 1 (<16.4)	Quartile 2 (16.4–59.7)	Quartile 3 (59.8–131.0)	Quartile 4 (≥131.1)
Vitamin C (mg)	Quartile 1 (<28.8)	Quartile 2 (28.8–53.6)	Quartile 3 (53.7–104.6)	Quartile 4 (≥104.7)
n-3 fatty acid (g)	Quartile 1 (<0.65)	Quartile 2 (0.65–1.23)	Quartile 3 (1.24–2.19)	Quartile 4 (≥2.20)
*Non-dietary*				
Smoking	Current (≥1 pack/day)	Current (<1 pack/day)	Former	Never
Alcohol intake	≥4 drinks/week	2–3 drinks/week	1–4 drinks/week	Never or <1 drink/month
Body mass index (kg/m^2^)	≥30	25–29.9	23–24.9	<23
Physical activity^a^	Quartile 1	Quartile 2	Quartile 3	Quartile 4

PUFA, polyunsaturated fatty acid.

a based on metabolic equivalent task (MET) values during work and leisure activities: 3.3 METs for walking, 4.0 METs for moderate activity, and 8.0 METs for vigorous activity.

Data on demographic characteristics (age, sex, and economic status) and comorbidities (physician-diagnosed hypertension, type 2 diabetes, and lung diseases [tuberculosis, asthma, and chronic obstructive pulmonary disease]) were also obtained. Mean family size–adjusted monthly income was used as an indicator of economic status, and the median value in the current sample was a cutoff-value for “high income” or “low income”.

### 2.3 Statistical analysis

Differences in median OB scores according to subject characteristics were compared using the Wilcoxon rank sum test. Odds ratios (ORs) and corresponding 95% confidence intervals (CIs) for reduced lung function per 1-point decrease in OB score were estimated by multivariable logistic regression analysis adjusted for potential confounders. Additionally, participants were categorized into 3 groups according to the level of FEV_1_ or FVC, and OB scores in each group were estimated using multivariate regression models. The analysis was performed using STATA MP Ver. 17.0 (Stata Corp., College Station, TX, USA). All statistical tests were two-sided, and *P* values < 0.05 were considered statistically significant.

## 3 Results

Table 2 presents the overall median OB scores and the comparison of median OB scores by subgroups. The median OB score was 17.0 points for all study subjects. Median OB scores were significantly lower in males and those with a low income, hypertension, type 2 diabetes, or reduced lung function (<80% of predicted FEV_1_ or FVC).

**Table 2:** Median oxidative balance scores according to subject characteristics.

Characteristics	No. of Subjects	Oxidative balance scores, median (IQR)	*P* values^c^
Overall	17,368	17 (15–19)	
Age			
<60 years	10,408	17 (15–19)	0.428
≥60 years	6,960	17 (15–19)	
Sex			
Male	7,992	16 (14–18)	<0.001
Female	9,376	18 (16–20)	
Income^a^			
High	9,884	17 (15–20)	<0.001
Low	7,445	17 (14–19)	
Hypertension			
No	12,489	17 (15–19)	<0.001
Yes	4,879	16 (14–19)	
Type 2 diabetes			
No	15,489	17 (15–19)	<0.001
Yes	1,879	16 (14–19)	
Lung disease^b^			
No	16,125	17 (15–19)	0.126
Yes	1,243	17 (15–19)	
Forced expiratory volume in 1-sec			
≥80% of predicted	13,815	17 (15–19)	<0.001
<80% of predicted	3,553	17 (14–19)	
Forced vital capacity			
≥80% of predicted	14,124	17 (15–19)	<0.001
<80% of predicted	3,244	16 (14–19)	

IQR, interquartile range.

a by median household income.

b includes pulmonary tuberculosis, asthma, and chronic obstructive pulmonary disease.

c analyzed using the Wilcoxon rank sum test.

Table 3 shows the OR and 95% CI for reduced lung parameter per 1-point decrease in OB scores. Overall, the association between oxidative imbalance and reduced lung function was more likely to be remarkable in FVC than in FEV_1_ (OR, 1.06 vs. 1.03; both p < 0.001). Nearly all subgroup except a few (i.e., FEV_1_ in female and patients with lung disease) reached the statistical significance.

**Table 3:** Odds for reduced lung parameter (<80% of predicted) per 1-point decrease in oxidative balance score.

		Forced expiratory volume in 1 s	Forced vital capacity	
	OR^c^ (95% CI)	*P* value	OR^c^ (95% CI)	*P* value
Overall	1.03 (1.02–1.04)	<0.001	1.06 (1.04–1.07)	<0.001
Age				
<60 years	1.03 (1.01–1.04)	0.002	1.05 (1.03–1.07)	<0.001
≥60 years	1.04 (1.02–1.06)	<0.001	1.06 (1.04–1.08)	<0.001
Sex				
Male	1.05 (1.04–1.07)	<0.001	1.06 (1.04–1.08)	<0.001
Female	1.00 (0.98–1.02)	0.762	1.05 (1.03–1.07)	<0.001
Economic status^a^				
High	1.03 (1.02–1.05)	<0.001	1.06 (1.04–1.08)	<0.001
Low	1.03 (1.01–1.05)	0.002	1.05 (1.03–1.07)	<0.001
Hypertension				
No	1.03 (1.01–1.04)	<0.001	1.05 (1.03–1.06)	<0.001
Yes	1.03 (1.01–1.05)	0.002	1.07 (1.05–1.09)	<0.001
Type 2 diabetes				
No	1.03 (1.02–1.04)	<0.001	1.06 (1.04–1.07)	<0.001
Yes	1.03 (1.00–1.07)	0.048	1.05 (1.02–1.08)	0.003
Lung disease^b^				
No	1.03 (1.02–1.04)	<0.001	1.05 (1.04–1.07)	<0.001
Yes	1.03 (0.99–1.07)	0.092	1.07 (1.02–1.11)	0.002

OR, odds ratio; CI, confidence interval.

a by median household income.

b includes pulmonary tuberculosis, asthma, and chronic obstructive pulmonary disease.

c adjusted for age, sex, economic status, and comorbidities (hypertension, type 2 diabetes, and lung disease).

**Figure 1:**
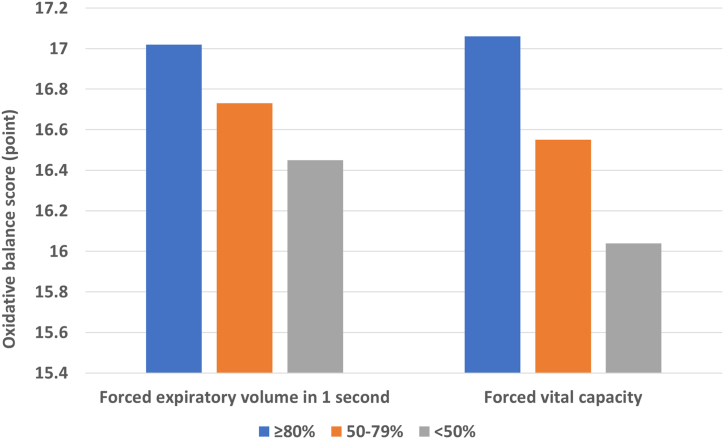
Association between severity of impaired lung function and oxidative balance score (p for trend<0.001 in both). Oxidative balance scores were estimated by multivariate regression analysis adjusted for age, sex, economic status, and comorbidities (hypertension, type 2 diabetes, and lung disease).

Figure 1 depicts the association between severity of lung function impairment and OB scores. Severely impaired group had the lowest OB scores in both FEV_1_ (OB score, 16.45 vs. 16.73 vs. 17.02) and FVC (OB score, 16.04 vs. 16.55 vs. 17.06), and significant linear relationships were noted (p for trend <0.001 in both).

## 4 Discussion

This nationally representative study identifies an association between reduced lung function and oxidative imbalance and vulnerable groups in which this association was most prominent. To our knowledge, only one previous study has examined this issue, and it reported a significant negative correlation between FEV_1_ and plasma oxygen free radicals in 30 healthy adults, which is consistent with our results [9]. Furthermore, a recent interventional study on antioxidant intake supports our findings [14].

Previous reviews [15, 16] have highlighted the importance of reactive oxygen/nitrogen species in the airway, as they inactivate antiproteases, induce apoptosis, regulate cell proliferation, and initiate inflammatory responses through transcription factor activation, signal transduction, chromatin remodeling, and induce the gene expressions of pro-inflammatory mediators. However, although antioxidants prevent or delay these processes, lung tissue damage may begin when the burden of oxidative stress exceeds antioxidant capacity.

OB score is a concept rather than a specific measurement, and researchers tend to use quite different scoring systems. Some have used only lifestyles or dietary factors as OB score components [17, 18], while others have utilized more integrated dietary components in combination with lifestyle components [19] and medications [20]. The OB scoring system used in this study, although not validated, was used in a previous KNHANES study [13]. We considered that validation per se was not important in the present study because it addressed the association between OB scores and lung function and not OB score cut-off values. However, we believe OB scores with antioxidant and pro-oxidant components would provide a simpler and more useful index in practice than specific markers [4].

Several limitations of this study should be considered. First, the cross-sectional nature of KNHANES precludes inferences regarding temporal relationships, and thus longitudinal studies are required to provide more definitive conclusions. Second, the OB Scoring System has several inherent limitations: 1) dietary factors are vulnerable to recall bias, 2) scores do not reflect endogenous factors, and 3) threshold effects of antioxidants are not considered [21]. Thus, we recommend that the study be replicated using biochemical measurements of oxidative stress. Third, the study population consisted of Korean adults, which limits the generalizability of our results.

Despite these limitations, this is the first epidemiologic study to investigate the association between oxidative balance and lung function in a general population. Our results suggest that oxidative stress can reduce lung capacity before overt respiratory disease develops. Interventional investigations and/or replicate studies in other ethnicities are warranted to confirm or refute our findings.

## Declarations

### Author contribution statement

Yoo Jeong Lee: Analyzed and interpreted the data; Wrote the paper.

In Cheol Hwang: Conceived and designed the experiments; Performed the experiments; Analyzed and interpreted the data; Contributed reagents, materials, analysis tools or data; Wrote the paper.

Hong Yup Ahn: Analyzed and interpreted the data; Contributed reagents, materials, analysis tools or data.

## Funding statement

Dr. In Cheol Hwang was supported by the 10.13039/501100006107Gachon University Gil Medical Center [FRD2021-14].

## Data availability statement

Data associated with this study has been deposited at The KNHANES web site (https://knhanes.kdca.go.kr/knhanes/eng/index.do).

## Declaration of interest's statement

The authors declare no competing interest.
